# Long-Term Changes in Weight in Patients With Severe and Complicated Obesity After Completion of a Milk-Based Meal Replacement Programme

**DOI:** 10.3389/fnut.2020.551068

**Published:** 2020-09-30

**Authors:** Razk Abdalgwad, Mohammed F. Rafey, Siobhan Foy, Micheál Newell, Colin Davenport, Derek T. O'Keeffe, Francis M. Finucane

**Affiliations:** ^1^Bariatric Medicine Service, Center for Diabetes, Endocrinology and Metabolism, Galway University Hospitals, Galway, Ireland; ^2^Health Research Board, Clinical Research Facility, National University of Ireland Galway, Galway, Ireland; ^3^Department of Medicine, National University of Ireland Galway, Galway, Ireland

**Keywords:** severe obesity, bariatric, meal replacement, weight regain, milk, dietary intervention

## Abstract

**Introduction:** Even with very significant short term weight loss with intensive dietary restriction, subsequent weight regain remains a challenge for most patients. We sought to assess long-term weight change in patients with obesity following completion of a 24-week milk-based meal replacement programme.

**Methods:** We conducted a retrospective cohort study of bariatric patients who completed our milk-based meal replacement programme. This programme started with an 8-week weight loss phase, followed by weight stabilization (8 weeks) and weight maintenance (8 weeks) phases, after which patients were followed up in the bariatric outpatient clinics. A paired sample t-test was used to compare mean differences in weight at the start and the end of the programme and at follow-up. Linear regression was used to identify predictors of weight regain.

**Results:** In total, 78 patients had long term follow-up data at a mean of 34.4 ± 19.8 months after the start of the milk diet and were included in this analysis. Mean body mass index at baseline was 50.5 ± 7.6 kg m^−2^, 41 (52.6%) were female and the mean age was 51.6 ± 12.0 (range 18.0–71.5) years. Weight decreased from144 ± 26 kg at the start of the milk diet to 121.2 ± 24 kg at completion (*P* < 0.001), with a non-significant trend upwards in the 1st and 2nd years of follow-up to 129.0 ± 27.7 (*P* = 0.07 compared to nadir) and 123.4 ± 29.0kg (*P* = 0.17), respectively. Although regains in the 3rd and 4th follow-up years were substantial to 131.0 ± 22.3 (*P* < 0.001), and 139.8 ± 35.4 kg (*P* < 0.001), there was still a moderate net weight loss of 4.7 [9.5, 0.21] and 7.0 [13.9, 0.26] kg (both *P* = 0.04) between the start and the 3rd and 4th follow-up years, respectively. The amount of weight regain was inversely associated with weight loss at completion of the programme, age, and directly associated with the duration of follow up in months (β = 1.2 [0.46, 1.9] *P* = 0.002).

**Conclusion:** In patients with severe obesity who completed a milk-based meal replacement programme and lost a large amount of weight, over 4 years of follow-up there was very substantial weight regain. Greater initial weight loss and older age were associated with less subsequent weight regain.

## Introduction

Obesity is a rapidly growing and highly prevalent multisystem disease (1). Obesity increases the risk of many co-morbid conditions (2, 3) that can decrease life expectancy (4), increase rates of disability (5), and impose significant economic burdens on patients and on society (6–8). Reducing weight via lifestyle, medical, or surgical interventions can lead to significant improvements in various markers of health in patients with obesity (9), but weight regain over time remains a significant challenge (10). Whether or not weight regain occurs following an intervention (and the degree to which it occurs) appears to vary depending on the specific intervention and the study population. A systematic review of 80 randomized clinical trials studying eight different types of non-surgical weight loss intervention reported that although short-term (6 months) weight loss varied from 5 to 16%, after an additional 6 months, significant weight regain occurred (11). Furthermore, in studies such as the SCALE trial, drugs like liraglutide help to maintain and even increase weight loss amongst those who have already achieved more than 5% weight loss with a low calorie diet, but once the drug is stopped, weight regain occurs within weeks (12). In contrast, bariatric surgery is associated with much less weight regain in comparison to lifestyle modification or drug interventions in the longer term (13), with the Swedish Obese Subjects (SOS) study describing maintenance of 18% total body weight loss after 20 years of follow-up after surgery compared to only 1% weight loss with non-surgical weight loss strategies (14). Ultimately, while the literature to date has identified weight regain as a significant problem, the data are somewhat conflicting, with results appearing to depend on the precise intervention utilized in particular.

In our own bariatric center in Galway University Hospitals (GUH) we provide a 24-week milk-based low-energy liquid diet (LELD) programme for patients with severe and complicated obesity. During this outpatient programme, patients are monitored intensively with fortnightly clinical assessments and blood tests. As our group has previously reported, during these 24 weeks patients typically demonstrate significant improvements in anthropometric and metabolic outcomes such as weight and HbA1c, with 86.7 and 48.6% of patients achieving ≥10 and ≥15% weight loss at 24 weeks, respectively (15). These results are consistent with those from other centers using equivalent interventions, such as was reported by a group in Cambridge who described a similar milk-based hypocaloric dietary intervention where 69% of participants who completed the intervention achieved ≥10% weight loss over a similar time period (16). What has yet to be investigated and reported, however, is the extent to which weight regain may occur in the aftermath of a milk-based hypocaloric meal replacement programme despite the magnitude of the weight loss observed in the short term. We sought to determine the longer-term changes in weight in patients who completed our intensive milk-based meal replacement programme and participated in our retrospective cohort study describing the short-term outcomes. Secondly, we sought to identify any factors associated with weight regain.

## Methods

This was a single-center long-term longitudinal retrospective cohort study of patients with obesity who completed all 24 weeks of our milk-based LELD programme. The study was conducted in the Diabetes Day Care Center (DDC) of GUH between January 2013 and October 2018. Inclusion criteria for our milk-based programme are as follows: Male and female patients with severe obesity (BMI ≥40 or ≥35 kg m^−2^ with co-morbidities such as type 2 diabetes, obstructive sleep apnoea syndrome or fatty liver disease) aged 18 years or older. The programme is not deemed suitable for pregnant or breast-feeding women or women intending to become pregnant or who are not using adequate contraceptive methods. Furthermore, patients with a recent myocardial infarction (within 6 months), untreated arrhythmias, untreated left ventricular failure, recent cholelithiasis (within the past year), hepatic, or renal dysfunction, type 1 diabetes, major psychiatric disorders, eating disorders, cancer, previous bariatric surgery, a BMI <35 kgm^−2^ or those deemed unlikely to attend for the full programme (e.g., frequent clinic non-attendance) are also not considered suitable candidates for the programme.

This retrospective study was approved by the Galway University Hospitals' Central Research Ethics Committee in December 2017 (ref CA 1900). As the programme was part of standard clinical care for patients attending our service between 2013 and 2018 and was not a prospective research study, we did not prospectively obtain written informed consent from patients to use their data for research purposes. However, with subsequent changes in European legislation regarding the use of personal data [the General Data Protection Regulation (GDPR)], we have only used data in this study from the subgroup of patients who agreed to this retrospectively and who provided written informed consent.

Our milk-based LELD programme consisted of three continuous 8-week phases as described in detail by our group previously (15). In brief, during the weight loss phase (weeks 1–8 inclusive) a milk-based liquid diet was prescribed consisting of ~2.5 L/day of semi-skimmed milk with additional sodium replacement, vitamin, mineral, and fiber supplementation, equating to ~1,200 kcal/day. The precise caloric content and volume of milk was determined by baseline body weight according to a departmental standard operating procedure. During the weight stabilization phase (weeks 9–16 inclusive) low calorie solid meals were gradually reintroduced from a set menu under the supervision of the bariatric dietitian. Finally, during the weight maintenance phase (weeks 17–24 inclusive), the milk component of the diet was stopped completely and a fully solid isocaloric diet was restarted, based on individualized meal plans compiled by the bariatric dietitian. During the intervention, patients attended our center every 4 weeks for 6 months, with fourteen visits in total. At each visit, patients had blood tests, weight measurements and met the nurse specialist, the bariatric dietitian and the bariatric physician.

Weight was measured with a Tanita® scale and height with a Seca® wall-mounted stadiometer, according to departmental standard operating procedures. For the purposes of the present study, data from these visits were retrospectively gathered on those patients who completed the entire programme, had follow-up visits in our center and who subsequently provided consent for their data to be gathered and analyzed for this purpose. Data were acquired from both paper-based medical charts and hospital electronic databases, including information on the latest available weight measurement for each patient after completion of the programme. We also recorded information on whether or not patients had subsequently had bariatric surgery at our center or elsewhere and excluded these patients from the analysis on the basis that surgical intervention would have a very substantial confounding effect on their subsequent weight trajectory (17, 18). Similarly, we identified whether patients had undergone treatment with weight loss medications and if so, excluded them from the analysis for the same reason. We did not record information on the extent to which patients participated in commercially available lifestyle modification programmes outside of the clinical care pathway provided at our clinic. Nor did we record information about over-the-counter obesity medication usage or the consumption of supplements and other products that are marketed as treatments for obesity. All patients attending our clinic after completion of the milk-based meal replacement programme were followed up individually on an annual basis with assessment by the specialist bariatric nurse, dietitian, and physician. Routinely, all patients attending our bariatric service complete a separate, 10-week, group-based structured lifestyle modification programme [Croi CLANN (Changing Lifestyle with Activity and Nutrition)] which we have described in detail previously (19). As the overall effect of this intervention on weight is modest (with a mean weight loss of 2.7 kg after 10 weeks), we did not compare outcomes in Croi CLANN completers separately in this analysis.

SPSS version 26 was used to carry out all statistical analyses. Descriptive statistics were used to interpret demographic characteristics of patients. Categorical variables were presented as numbers and percentages, while continuous variables were presented as means and standard deviations for normally distributed data, and medians and interquartile ranges (IQR) for non-normally distributed data. As the duration of follow-up varied so widely, we categorized patients into four groups according to how long (after completion of the milk-based programme) we had follow-up weight and clinical data available on them to enter in the analysis. Group 1 included those patients for whom follow-up weight data was available from between 6 and 18 months after completing the programme. Group 2 included those patients for whom follow-up data was available from between 18 and 30 months after completing the programme. Group 3 included patients with follow-up data from 30 to 42 months after the programme, and Group 4 included those with data available after 42 months of follow up.

To compare categorical variables between the four groups, Pearson's chi-square test was used. Non-normally distributed variables were compared using the nonparametric median test. The paired sample *t*-test was used to compare the mean differences in weight at the start and at completion of the milk programme, and from completion to follow up. We calculated the relative percentage of weight regain by expressing the amount of weight regained in the follow-up period as a percentage of the amount of weight lost during the intervention, using the formula:

*Relative percentage weight regain* = *(Weight at follow-up – Weight at milk programme completion)/(Weight at milk programme start – Weight at milk programme completion)* × *100*.

A multivariate linear regression analysis was used to determine predictors of weight regain. We used the relative percentage weight regain as the dependent (or outcome) variable, and the independent variables of sex, age, and total weight loss percentage (TWL%) after 24 weeks of the milk diet. Additionally, we conducted a stepwise backward multivariate linear regression model to determine the best predictors of weight regain.

## Results

Between January 2013 and October 2018, 260 patients were enrolled in the milk-based meal replacement programme at the Bariatric Medicine Clinic in Galway University Hospitals. Of these, 139 (53.5%) completed all 24 weeks of the intervention, with 121 (46.5%) discontinuing the intervention. Of the 139 completers, 105 (75.5%) agreed to participate in this study and provided written informed consent. Given that 1,867 new patients were seen in our bariatric service over the 6-year study period we note that 13.9% of newly referred bariatric patients ultimately participated in our milk programme (15). Of the 105 patients who consented to data gathering and retrieval, 78 were included in the final study population. We excluded from the analysis 17 patients who received GLP 1 therapy, 8 patients who received sleeve gastrectomy and 2 patients who received both. Of those 78 patients, 41 (52.6%) were female. The mean age was 51.6 ± 12.0 years. At baseline the entire cohort had a mean weight of 144 ± 26 kg and a BMI of 50.5 ± 7.6 kg/m^−2^. Of the 78 patients, 49 had attended the Croi CLANN programme (27 before starting the milk diet and 22 after completing the diet).

The mean follow-up period for the entire cohort was 34.3 ± 19.8 months. The number of individuals in each of the four follow-up duration subgroups is shown in Table 1. Only the most recently available weight measurement was used, so each patient is included in only one of the follow-up time periods, rather than several periods. The initial change in weight during the milk-based programme (from baseline to 24 weeks) in the 1st, 2nd, 3rd, and 4th year of follow-up was −20.2 [−23.3, −17.0], −26.5 [−33.3, −19.6], −20.6 [−24.4, −16.9], and −23.2 [−26.9, −19.4] kg, respectively (all *P* < 0.001).

**Table 1:** Baseline participant characteristics and changes in weight during and after the milk diet.

**Follow-up Group**	***N* (%)**	***N* (%) Female**	**Follow-up duration (Months)**	**Weight at Baseline**	**Weight at milk-diet completion**	**Weight at follow-up**	**Weight regain after milk diet completion^**†**^**	***P*^**†**^**	**Net weight change since start of milk diet^**‡**^**	***P*^**‡**^**
**6–18 months**	23 (29.5%)	12 (52.2%)	12.3 ± 3.7	146.0 ± 26.1	124.9 ± 24.2 kg	129 ± 27.7 kg	4.0 [−0.43, 8.5] kg	0.07	−17.0 [−22.5, −11.5] kg	<0.001
**18–30 months**	14 (17.9%)	8 (57.1%)	23.5 ± 3.0	142.4 ± 25.5	115.9 ± 20.3 kg	123.4 ± 29.0 kg	7.5 [−3.8, 18.9] kg	0.17	−18.9 [−33.7, −4.1] kg	0.01
**30–42 months**	12 (15.4%)	7 (58.3%)	36.8 ± 4.0	135.9 ± 21.3	115.2 ± 21.5 kg	131.0 ± 22.3 kg	15.7 [9.8, 21.6] kg	<0.001	−4.7 [−9.5, −0.21] kg	0.04
**>42 months**	29 (37.2%)	14 (48.3%)	56.0 ± 11.0	146.8 ± 27.6	123.6 ± 26.4 kg	139.8 ± 35.4 kg	16.1 [9.9, 22.3] kg	<0.001	−7.0 [−13.9, −0.26] kg	0.04

*The paired sample t test was used to report mean differences in weight between completion of the milk diet and follow-up, and between the start of the milk diet and follow-up*.

*Denotes the change in weight from completion of the milk diet to subsequent follow-up (mean and [95% confidence interval])*.

‡ *Denotes the net change in weight from the start of the milk diet to subsequent follow-up (mean and [95% confidence interval])*.

In the 1st and 2nd years after the completion of the programme some weight regain was observed but this did not achieve statistical significance. The mean difference between weight at completion of the programme and at 1-year post-completion was 4.0 [−0.43, 8.5] kg, *P* = 0.07. Similarly, the mean difference between weight at completion of the programme and weight at 2 years was 7.5 [−3.8, 18.9] kg, *P* = 0.17. However, although weight regain in the 3rd, and 4th years proved to be substantial, at 15.7 [9.8, 21.6] kg (*P* < 0.001) and 16.1 [9.9, 22.3] kg (*P* < 0.001), there was still a moderate net weight loss of 4.7 [9.5, 0.21], and 7.0 [13.9, 0.26] kg (both *P* = 0.04), respectively, compared to the start of the programme (Table 1).

**Figure 1:**
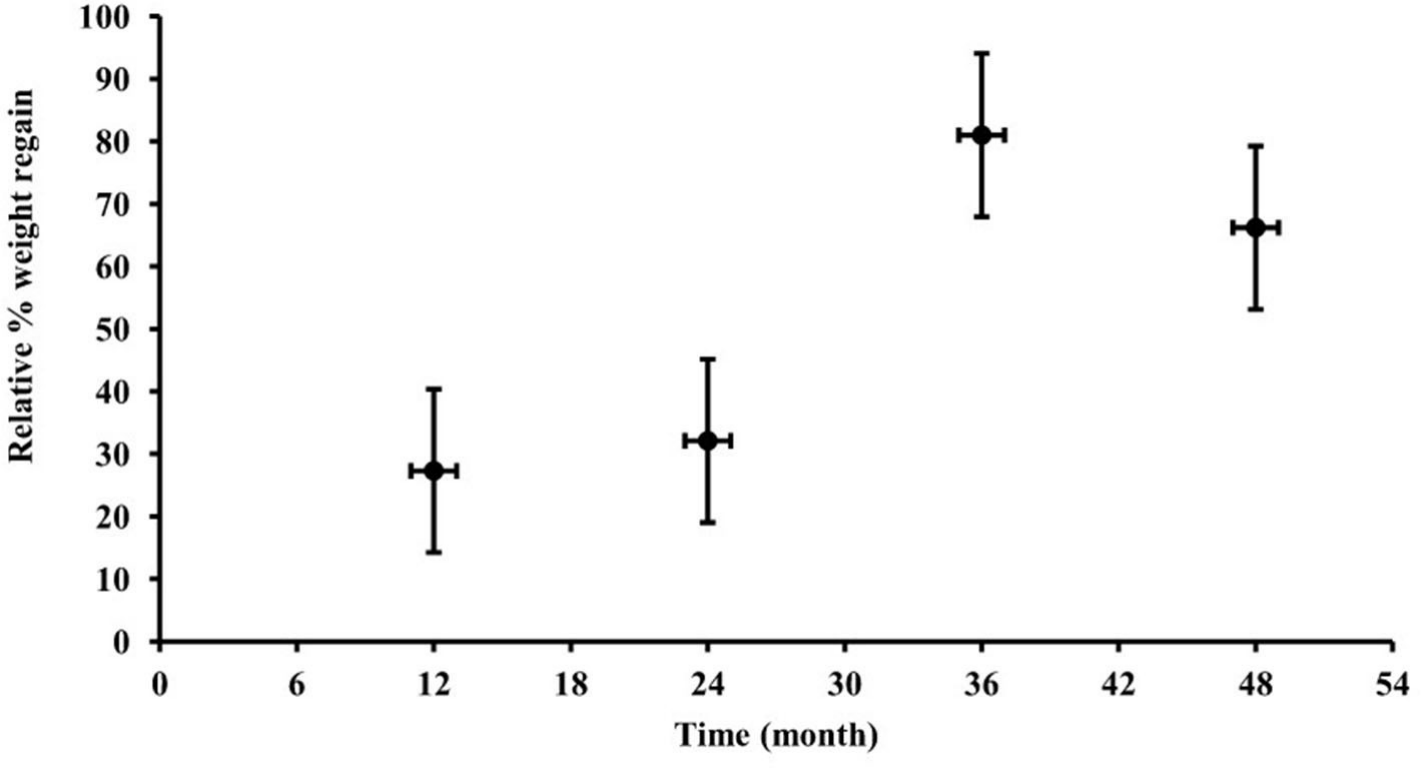
Relative percentage weight regain in milk diet completers (from completion of the intervention at 6 months) in participants followed in the 1st, 2nd, 3rd, or 4th year. Percentages of weight regain are presented as medians and interquartile ranges (vertical error bars) while horizontal error bars depict the standard deviations of time to follow-up (in months).

The median percentage relative weight regain in the 1st, 2nd-, 3rd-, and 4th-year follow-up groups was 27.3 [−12.1, 60.2], 32.1 [3.3, 94.3], 81.0 [38.4, 99.6], and 66.2 [29.1, 125.2]%, respectively (*P* = 0.05), as shown in Figure 1. Put another way, while 68 of 78 patients (87.2%) achieved ≥10% weight loss during the milk programme, only 43.5, 42.9, 8.3, and 31.0% of patients maintained ≥10% weight loss in the 1st, 2nd, 3rd, and 4th year of follow-up, respectively.

In multivariate regression modeling to identify potential predictors of weight regain, rather than categorizing the duration of follow-up, we treated it as a continuous variable (measured in months) in the model. With this, we observed a statistically significant inverse association between TWL% at the end of the milk diet and subsequent relative weight regain, both in analyses adjusted only for the duration of follow-up, as well as analyses additionally adjusted for age and sex, as shown in Table 2. Age was also inversely associated with percentage relative weight regain, adjusting only for duration of follow-up and also adjusting additionally for sex (Table 2).

**Table 2:** Analysis of Potential Predictors of Relative Weight Regain after Completion of the Milk-Based Meal Replacement Programme.

**Variable**	**β**	**95% CI**	***p***
TWL% (at 24 weeks)^*^	−3.3	[−5.7, −0.9]	0.009
TWL% (at 24 weeks)^**^	−3.2	[−5.4, −1.0]	0.004
Age (years)^*^	−2.1	[−3.3, −1.0]	<0.001
Age (years)^***^	−2.0	[−3.2, −0.9]	0.001
Duration of follow-up	1.1	[0.4, 1.9]	0.005
Duration of follow-up^****^	1.2	[0.5, 1.9]	0.002

*All analyses refer to multivariate linear regression modeling with the percentage relative weight regain being the dependent variable*.

* *Denotes analyses adjusted only for duration of follow-up (in months)*.

** *Denotes analyses adjusted for duration of follow-up, age, and sex*.

*** *Denotes analyses adjusted for duration of follow-up and sex*.

**** *Denotes analyses adjusted for age and sex*.

## Discussion

As many patients who suffer from obesity will confirm, it is frequently not loss of weight that is the main challenge they face in managing their disease but instead, it is maintaining that weight loss over the longer-term. The present study highlights that difficulty even in the aftermath of very significant weight loss achieved through a structured milk-based LELD programme with very frequent contacts between patients and the bariatric team. Over a follow-up period of 4 years, our data indicate that substantial weight regain occurred amongst our participants, albeit with persisting net weight loss. To the best of our knowledge, the present study is one of the few to provide long-term data after an effective weight loss intervention in patients with severe and complicated obesity and it is the first to examine weight regain after a milk-based LELD programme.

As noted previously, our study is consistent with some if not all of the previous research into long-term weight regain, and highlights the observation that dietary interventions (even when intensive and highly effective from the initial weight loss perspective) do not match bariatric surgery results with regards to the maintenance of weight loss (11, 12, 14). The reason for weight regain after successful weight loss is complex and likely multifactorial, with factors such as hypothalamic-mediated weight homeostasis, environmental and psychological variables, frequency of interactions with health care professionals, participation in exercise, and underlying genetic pre-dispositions of the patients themselves to obesity all playing potential roles (20–23). Studies in rats have confirmed a strong and persistent neuro-humoral response to caloric restriction that drives weight regain once ad libitum food intake is restored (24). These physiological counter-regulatory mechanisms to preserve energy balance are driven by genetic and epigenetic factors which lead to an imprinted obesogenic “memory” that prevents weight loss maintenance (25). In humans, very low calorie diet- induced weight loss leads to alterations in gut hormones that promote hunger and decrease satiety, ultimately driving weight regain (26, 27). Similar findings have been described with other dietary and exercise interventions and after bariatric surgery (28).

In terms of what can be done to counteract these physiological factors promoting weight regain, studies have suggested that the long-term adoption of behaviors that promote reducing energy intake and increasing energy expenditure (particularly self-monitoring of weight and eating, and cognitive/psychological behaviors such as self-efficacy for weight management and self-efficacy for exercise) may help to avoid regain (29, 30). Long-term behavior change, however, may be difficult to achieve, thus the problem of weight regain remains significant within the field of obesity.

With regards to predictors of weight regain within the present study, we report that patients who lost larger amounts of their total body weight saw less regain in follow-up. Whether this is a behavioral phenomenon (e.g., more observed weight loss motivated patients to a greater degree) or whether those patients were different at baseline in terms of their ability to lose weight and maintain said weight loss cannot be determined from the present study, but our results do suggest that greater initial weight loss may be associated with better weight loss maintenance. These findings are similar to observations from the National Weight Control Registry (NWCR) where those patients with greater initial reductions in body weight also saw better weight loss maintenance over the subsequent 10 years (31). Thus our findings are consistent with those previously reported, albeit in the novel setting of the aftermath of a milk-based LELD programme, and suggest that greater initial weight loss (and older age) during interventions may be positive long-term indicators.

Our study has a number of important limitations. Because of the retrospective design, bias and confounding may have been introduced which would be less likely in a prospective cohort study or a randomized controlled trial. The Variable duration of follow-up is another limitation. The use of groups based on the time period of their follow-up data provided a more accurate picture of weight regain over time when compared against a single average follow-up date for the entire cohort. However, this also led to smaller sample sizes for analysis. Therefore, it may be difficult to generalize the results from this study to other patient populations outside of a hospital-based cohort of patients with severe and complicated obesity. This limitation needed to be addressed in more robust large prospective study or randomized control trail in future. Furthermore, we note that most of patients in this cohort also went through our Croi CLANN lifestyle modification programme either before or after their participation in the milk-programme (19), and it is possible that participation in this programme (which focuses on sustainable lifestyle change and modest weight loss) may have helped to decrease weight regain in the aftermath of the milk programme (the mean weight loss in Croi CLANN study was 2.7 Kg) (19). This speculation, however, does not detract from our primary observation that weight regain to baseline occurred by 4 years in our population. Finally, we excluded patients who received GLP 1 agonist therapy or bariatric surgery after completing the milk programme, in an attempt to minimize the confounding effect of these interventions on the likelihood of weight regain. The decision to use these treatments was based on individual patient preference and on careful individualized clinical assessment, an analysis of which is beyond the scope of this paper. However, it is possible that worse weight regain may have prompted more intensive and invasive intervention with drugs or surgery, and that by excluding these patients from our analysis we have in fact underestimated the magnitude of the problem of weight regain after the milk diet. On balance we feel that excluding them from the analysis is the approach least likely to yield spurious results.

To conclude, among patients with severe and complicated obesity who completed a milk-based meal replacement programme, significant initial weight loss was followed by substantial weight regain. Weight regain was inversely associated with age and with total weight loss during the milk diet, and positively associated with the duration of follow up. It is clear that weight regain remains a significant challenge in the treatment of the chronic disease of obesity.

## Data Availability Statement

The raw data supporting the conclusions of this article will be made available by the authors, without undue reservation.

## Ethics Statement

The study was approved by the Galway University Hospitals' Central Research Ethics Committee in December 2017 (ref CA 1900). All of the participants provided written informed consent to participate in this study.

## Author Contributions

RA contributed to the study design, conducted data analysis, and prepared the manuscript. MR contributed to the study design, data collection, conducted data analysis, and prepared the manuscript. SF helped with data collection, intervention delivery, and revising the manuscript. MN, CD, and DO'K contributed to the data analysis and revised the manuscript. FF conceptualized the study design, led the clinical intervention and supervised the data collection, drafting of the manuscript, and the data analysis. All authors have given final approval of the version to be published and agree to be accountable for all aspects of the work.

## Conflict of Interest

CD, DO'K, and FF have received honoraria, travel grants, and have served on advisory boards for Novo Nordisk, Eli Lilly, Ethicon, Pfizer Inc., Sanofi-Aventis, Astra Zeneca, Merck-Serono, Boehringer Ingelheim, Janssen, and Novartis. The remaining authors declare that the research was conducted in the absence of any commercial or financial relationships that could be construed as a potential conflict of interest.

## Abbreviations

BMI: Body Mass Index
Croi CLANN: Changing Lifestyle with Activity and Nutrition
DDC: Diabetes Day Care Center
GUH: Galway University Hospitals
GDPR: The General Data Protection Regulation
IQR: Interquartile Range
LCD: Low Calorie Diet
LELD: Milk-Based Lowe-Energy Diet
NWCR: National Weight Control Registry
SOS: Swedish Obese Subjects study
SPSS: Statistical Package for the Social Sciences
TWL%: Total weight loss percentage.
